# Second Language Ability and Emotional Prosody Perception

**DOI:** 10.1371/journal.pone.0156855

**Published:** 2016-06-02

**Authors:** Anjali Bhatara, Petri Laukka, Natalie Boll-Avetisyan, Lionel Granjon, Hillary Anger Elfenbein, Tanja Bänziger

**Affiliations:** 1 CNRS (Laboratoire Psychologie de la Perception, UMR 8242), Paris, France; 2 Université Paris Descartes, Sorbonne Paris Cité, Paris, France; 3 Department of Psychology, Stockholm University, Stockholm, Sweden; 4 School of Social Sciences, Södertörn University, Huddinge, Sweden; 5 Department of Linguistics, Universität Potsdam, Potsdam, Germany; 6 Olin Business School, Washington University, Saint Louis, MO 63130, United States of America; 7 Department of Psychology, Mid Sweden University, Östersund, Sweden; Max Planck Institute for Human Cognitive and Brain Sciences, GERMANY

## Abstract

The present study examines the effect of language experience on vocal emotion perception in a second language. Native speakers of French with varying levels of self-reported English ability were asked to identify emotions from vocal expressions produced by American actors in a forced-choice task, and to rate their pleasantness, power, alertness and intensity on continuous scales. Stimuli included emotionally expressive English speech (emotional prosody) and non-linguistic vocalizations (affect bursts), and a baseline condition with Swiss-French pseudo-speech. Results revealed effects of English ability on the recognition of emotions in English speech but not in non-linguistic vocalizations. Specifically, higher English ability was associated with less accurate identification of positive emotions, but not with the interpretation of negative emotions. Moreover, higher English ability was associated with lower ratings of pleasantness and power, again only for emotional prosody. This suggests that second language skills may sometimes interfere with emotion recognition from speech prosody, particularly for positive emotions.

## Introduction

Listeners can often correctly infer the emotional state of a speaker—even if they do not understand the language that is being spoken—by using paralinguistic information included in the utterance. Numerous studies have demonstrated the above-chance recognition of emotions communicated through the voice across highly disparate cultures. This includes comparisons as culturally distant as American English speakers and listeners from the Shuar hunter-horticulturalists of the Amazonian Ecuador [1] and from remote villages in Bhutan [2], or mutually among speakers and listeners from England and the seminomadic Himba group in northern Namibia [3].

Although emotions are reliably recognized across cultures, many studies have shown an in-group advantage, meaning that native speakers of the language of testing or within-culture group members typically perform better than those outside of the language or culture [4,5]. To explain this phenomenon, Elfenbein & Ambady [6] proposed the dialect theory, suggesting that the way emotions are communicated varies subtly across groups—similar to dialects in language—so that there are both universal and culture-specific aspects of the emotion display that modulate emotion perception. The current study aims at investigating these culture-specific aspects, and whether the perception of emotion in a second language (“L2”) is affected by knowledge of this L2. To investigate this, participants' perception of emotion in both linguistic (utterances containing emotional prosody) and non-linguistic stimuli (affect bursts) is measured, with the hypothesis that linguistic experience should affect perception of (linguistic) emotional prosody but not affect bursts.

Few previous studies have examined L2 learners' emotion perception in their L2, and results are mixed. The main task in all of the studies below was to identify emotions or attitudes from emotional prosody by choosing response alternatives from lists provided by the experimenters. Of the studies that have compared L2 learners with native listeners, two showed that L2 learners recognized emotions in that L2 less well than native listeners [4,7], one showed that L2 learners performed less well than polyglot native listeners but did not differ from monolingual native listeners [8], and one showed the opposite pattern, with L2 learners outperforming native listeners [9]. The language tested in the last study [9] was Mandarin, learned by native Dutch listeners, and the author proposes that the counterintuitive findings are due to the fact that Mandarin uses tonal information both grammatically and paralinguistically. This may give listeners whose native language is non-tonal an advantage, as they can more easily ignore the grammatical meaning of the tonal information to focus on its prosodic aspects.

In the studies comparing groups with and without L2 experience, there have often been confounding factors and/or mixed results. Altrov [7] showed higher performance among listeners who had L2 experience compared with those who did not, but the two groups also lived in different countries, so it is difficult to separate the influences of cultural and linguistic experience. Similarly, Shochi, Gagnié, Rilliard, Erickson, and Aubergé [10], testing forced-choice recognition of social attitudes, showed an advantage among L2 learners relative to those with no knowledge of the L2. However, these learners had received training on how to recognize emotions in their L2, and so it is difficult to disentangle the potential influence of these two forms of learning. Zhu [9] did show that listeners with L2 knowledge of the target language performed better than non-native listeners, implying that language experience can help with recognition of emotions from prosody. However, Graham et al. [4] showed no difference between high- and low-proficiency L2 learners.

When examining the effect of knowledge on emotion perception, it is important to keep in mind that different emotions may differ in their universality of expression. This may provide a possible means of explaining previous conflicting results in studies that focused on perception across multiple emotions. In a meta-analysis, Elfenbein & Ambady [11] showed that, across studies examining perception of emotion in the voice, anger and sadness were the most reliably recognized emotions cross-culturally, whereas happiness had the highest in-group advantage and thus was least reliably recognized across cultures (no other positive emotions have been studied enough to have been included in the meta-analysis). Along these same lines, Sauter et al. [3] showed that non-verbal vocal sounds reliably communicated a set of primarily negative emotions across cultures, but not necessarily more positive emotions. Positive emotions may be more culturally specific than negative emotions: if a function of positive emotion is to facilitate group cohesion, this might be why they are more recognizable to in-group members than to outsiders, with whom group cohesion is not as important and may even be undesirable [3]. Similarly, Ekman [12] suggests that they may be evolutionarily less important than negative emotions. By extension, we postulate that positive emotions may be communicated in a more linguistically specific way. That is, positive emotions might use prosodic cues that have a relatively greater advantage for native speakers of that language to interpret. In addition, expression of positive emotions has not been studied as thoroughly as for negative emotions; most of the "basic" emotions, those that have been most often examined, are negative [13]. Perhaps, in addition to the distinction between basic and complex emotions, it is worthwhile to focus on the distinction between positive and negative emotions. In the present study, we investigate the extent to which proficiency in a second language has effects on the perception of vocal affect bursts and/or emotional prosody uttered in this second language. To examine this, we tested French native listeners' perception of American English vocal expressions. Note that all of the papers investigating L2 perception of emotion that were discussed in the summary above included a majority of negative emotions in their stimulus sets. Most included only one positive emotion (joy), and two of the studies [8,9] also included "surprise," but neither specified it as positive surprise. In the present study, our goal was to investigate the influence of proficiency on positive and negative emotions separately, so we required a balanced number of each. Hence, we contrasted four negative emotions (fear, anger, sadness, and disgust) and four positive emotions (pride, interest, joy, and relief), each expressed in both vocal affect bursts and emotional prosody.

Specifically, we strive to answer three questions: First, is English L2 ability related to recognition and/or rating of emotion in vocal expressions? Second, does this relation depend on whether the expressions are emotional prosody or affect bursts (i.e., linguistic or not)? Third, does this relation differ depending on whether emotions are negative or positive? To answer these questions, French participants are presented with American English emotional prosody and affect bursts and asked to select the emotion expressed and to rate the stimulus on four continuous scales (described below). To our knowledge, no prior study investigating L2 experience and emotion perception has included continuous measures. The inclusion of these measures may allow us to capture more fine-grained differences in emotion perception, for example within emotion categories, that could be affected by language experience. To control for inter-individual differences in emotion perception, we include emotional prosody stimuli spoken by native speakers, providing baseline measures of their emotion-identification ability.

## Method

### Participants

A group of 48 native French-speaking adults (21 M, 27 F, mean age = 23, age range = 18–33) completed this task. All research was conducted according to the principles expressed in the Declaration of Helsinki. Written informed consent was obtained from every participant, and the study was approved by the university's ethics review board (*Comité d’évaluation éthique des projets de recherche en santé*), #2012–05. All participants had learned English in school, starting between the ages of 6 and 15 (*M* = 9.8 years) for 2–17 years (*M* = 9.7 years).

### Stimuli

Participants were presented with utterances or vocalizations produced with eight different emotions (anger, disgust, fear, sadness, interest, pride, relief, and happiness). Each participant completed two experimental and one control condition. In the first experimental condition, stimuli were 48 English sentences spoken by American actors from the Vocal Expressions of Nineteen Emotions across Cultures (VENEC) corpus (“That’s exactly what happened,” and “Let me tell you something.”). In the second condition, stimuli were 48 nonverbal vocalizations (e.g., crying, hums, grunts, laughter) from the VENEC corpus, also produced by the same American actors. In the control condition, participants heard 24 pseudo-French utterances spoken by Swiss actors from the Geneva Multimodal Emotion Portrayals (GEMEP) corpus (“Ne kali bam sud molen!” and “Kun se mina lod belam?”). For more details on the recording and validation of these stimuli, see [14, 15,16].

### Procedure

Stimuli were presented using E-prime 2 through Sennheiser HG 558 headphones. Participants gave written informed consent and then completed the three conditions in counterbalanced order. For each trial in each condition, they heard an utterance or vocalization and gave five responses: the first was a forced-choice task to judge (via a mouse-click) which emotion out of eight displayed on the screen they thought was being expressed by the voice. The emotion words (in French: *colère*, *dégoût*, *fierté*, *intérêt*, *joie*, *peur*, *soulagement*, *tristesse*) were randomly rearranged on the screen at the beginning of each condition, but remained in place throughout that condition. The other four responses were to rate the voice using a continuous slider scale on four dimensions: 1) emotion intensity, ranging from *émotion légère* (weak emotion) to *émotion forte* (strong emotion); for example, a person can be slightly angry (irritated) or very angry (furious); 2) pleasantness, ranging from *émotion désagréable* (unpleasant emotion) to *émotion agréable* (pleasant emotion); 3) power, ranging from *personne faible* (weak person) to *personne puissante* (powerful person); specifically, participants were asked to evaluate the speaker’s level of dominance, that is, control of the situation; and 4) activation, ranging from *personne assoupie* (sleepy person) to *personne attentive* (alert person). There were no numbers displayed on the sliders, but responses were recorded as scores between 0 and 100, with 100 indicating more intense, pleasant, powerful, or active. A translation of the full instructions given to the participants is available in the supplementary materials.

Following the experimental tasks, they filled out a questionnaire about their experience with English, including several self-ratings of their written and oral English ability and written and oral French ability on a scale of 0 to 10. Three of the self-rating questions (reading, listening comprehension, and speaking) were from the Language Experience and Proficiency Questionnaire (LEAP-Q, [17]) and the other four measures were added to increase precision (writing, grammar, pronunciation, and vocabulary). The questionnaire was used in previous studies [18,19]. These self-ratings are strongly correlated with various standardized proficiency tests (e.g., [17,19]) and Marian et al. [17] concluded that, compared with standardized measures of proficiency, “self-reported proficiency may be a more efficient way of indexing overall language performance, with language history information augmenting predictions of performance on specific linguistic tasks” (p. 960).

## Data Processing, Analysis & Results

### Data processing and analysis

**Fig 1:**
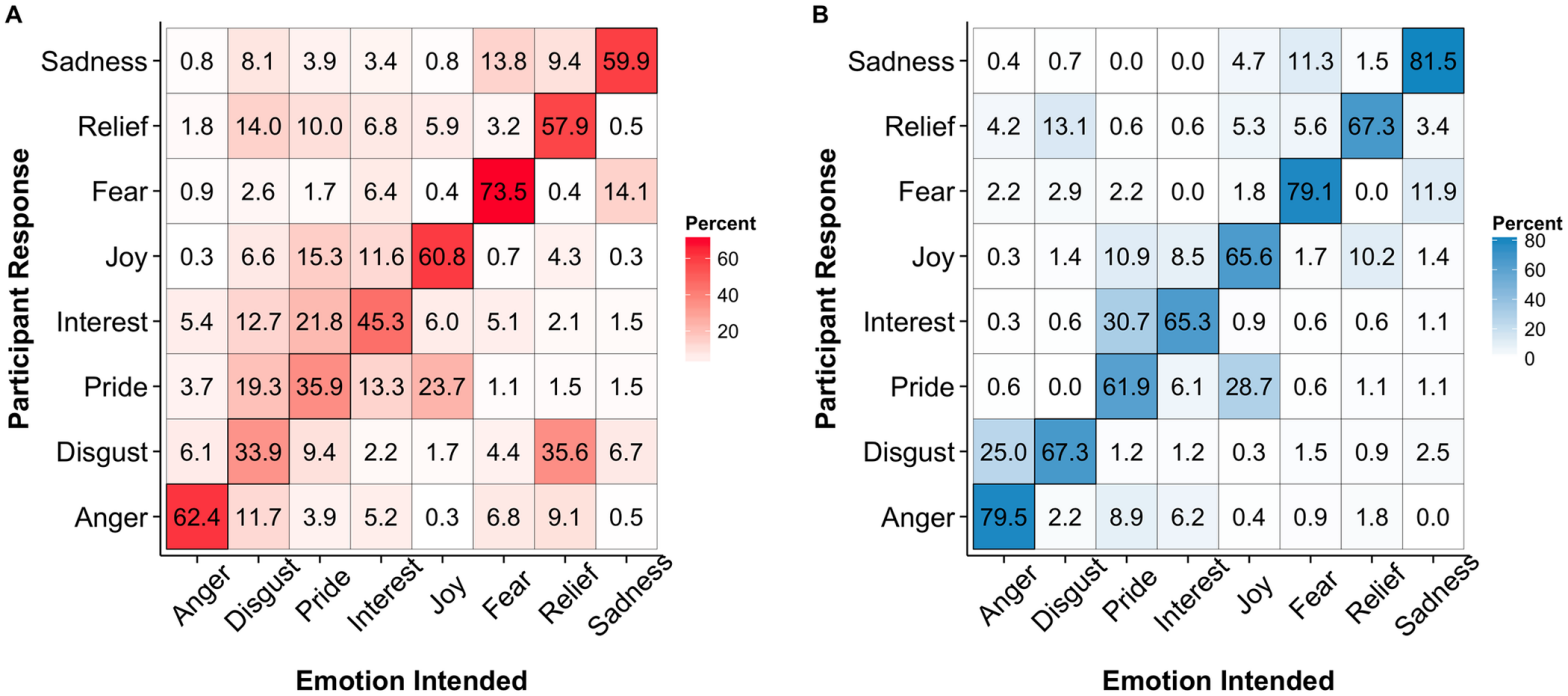
Confusion matrices for a) emotional prosody and b) affect burst tasks. Percentages indicate percent of responses given for each intended emotion, i.e., 14% of the time participants gave the response “relief” in the emotional prosody condition, the intended emotion was “disgust.”

The data from the two experimental conditions (affect bursts and emotional prosody) were analysed separately in generalized linear mixed logit models [20] fitted using the statistics program R [21] and the packages lme4 for analysis [22] and ggplot2 for figure creation [23]. See Fig 1 for confusion matrices for the two experimental conditions.

For each of the conditions, two types of analyses were carried out: one using the dependent variable *correct response*, and the other using the four continuous measures as dependent variables. For the analyses of the correct responses, we included three fixed factors: English ability, GEMEP score, and valence of the emotional expression judged. Principal Component Analysis was used to extract one variable for *English ability* that combined the self-rating scores of the seven questions on English ability mentioned above (See Table 1 for results). The first principal component (capturing 78% of the information contained in the seven separate variables) was used in this analysis. This continuous predictor was centered around its mean to reduce collinearity. *GEMEP score* reflects the results from the control condition with the pseudo-French stimuli, specifically, the proportion of correctly identified stimuli for each participant. This variable serves as a measure of baseline emotion identification ability for each participant in the prosody of their native language. The factor *valence* reflects a division of the emotions into positive (happiness, interest, pride, and relief) and negative (anger, disgust, fear, and sadness) groups.

**Table 1:** L2 self-rating (from 0 "none" to 10 "perfect").

Factor	Mean (SD)	Range
Vocabulary	5.3 (2.0)	1–8
Grammar	5.0 (2.2)	1–9
Writing	5.8 (1.9)	1–9
Reading	6.5 (1.8)	2–9
Speaking	5.6 (1.9)	1–9
Understanding spoken English	6.1 (1.9)	1–9
Pronunciation	5.3 (2.2)	1–9

The model included the main effects of each fixed factor as well as the interactions between self-reported English ability and valence and between valence and the GEMEP score. Examining the English ability × valence term allows us to address the second research question posed above, namely the extent to which the effect of English ability varies as a function of emotion. Valence was analysed using a successive differences contrast (the contrast.sdif function in R). The maximal model was performed [24], which included a random slope by subject for the valence and random slopes by stimulus for both English ability and the GEMEP score. However, this solution did not converge. Effects were gradually reduced until the maximal model that did converge included the interaction between English ability and valence, the interaction between the GEMEP score and valence, the random slope of valence by subject, and the random slope of English ability by stimulus. Binomial tests were performed for each emotion to ascertain whether they were correctly identified more often than chance. Linear mixed-effects models were also run on the four continuous measures, with the same effects structure as above, minus the GEMEP score.

### Results of the emotional prosody condition

Results of the analysis of the correct responses showed a significant main effect of GEMEP score (*p* = .004) and a significant interaction between valence and English ability (*p* = .007). No other factors or interactions were significant (all *p*s > .23. These results are summarized in Table 2. Note that *correct response* is a dichotomous variable (i.e., correct or no), so z-scores are reported. The binomial tests on each emotion (corrected for multiple comparisons) showed that all were identified at greater than chance levels (see Table 3).

**Table 2:** Results from emotional prosody condition.

	*β*(Estimate)	Standard Error	*z*-value	Probability > |*z*|
(Intercept)	-1.30	0.57	-2.27	.02
Valence	0.49	1.05	0.47	.64
English ability	-0.02	0.03	-0.56	.58
GEMEP score	2.19	0.77	2.85	.004
Valence * English ability	-0.14	0.05	-2.69	.007
Valence * GEMEP score	-1.66	1.40	-1.20	.23

**Table 3:** Results from binomial tests.

	Percent correct within emotion intended	Confidence Interval (%)	Percent correct within emotion intended	Confidence Interval (%)
	Emotional prosody condition		Vocal affect bursts condition	
Disgust	21.2	16.7–26.4	76.2	70.9–81.0
Pride	34.6	28.2–39.5	39.4	33.7–45.4
Interest	52.1	46.1–58.0	80.4	75.3–84.9
Fear	59.7	53.8–65.4	76.8	71.5–81.6
Joy	63.5	57.7–69.1	67.2	61.5–72.6
Sadness	79.9	74.8–84.3	78.0	72.8–82.7
Anger	83.0	78.1–87.1	62.7	56.8–68.3
Relief	44.4	38.6–50.4	84.3	79.5–88.3

Note: All significantly different from chance (12.5% correct) at *p* < .001

**Fig 2:**
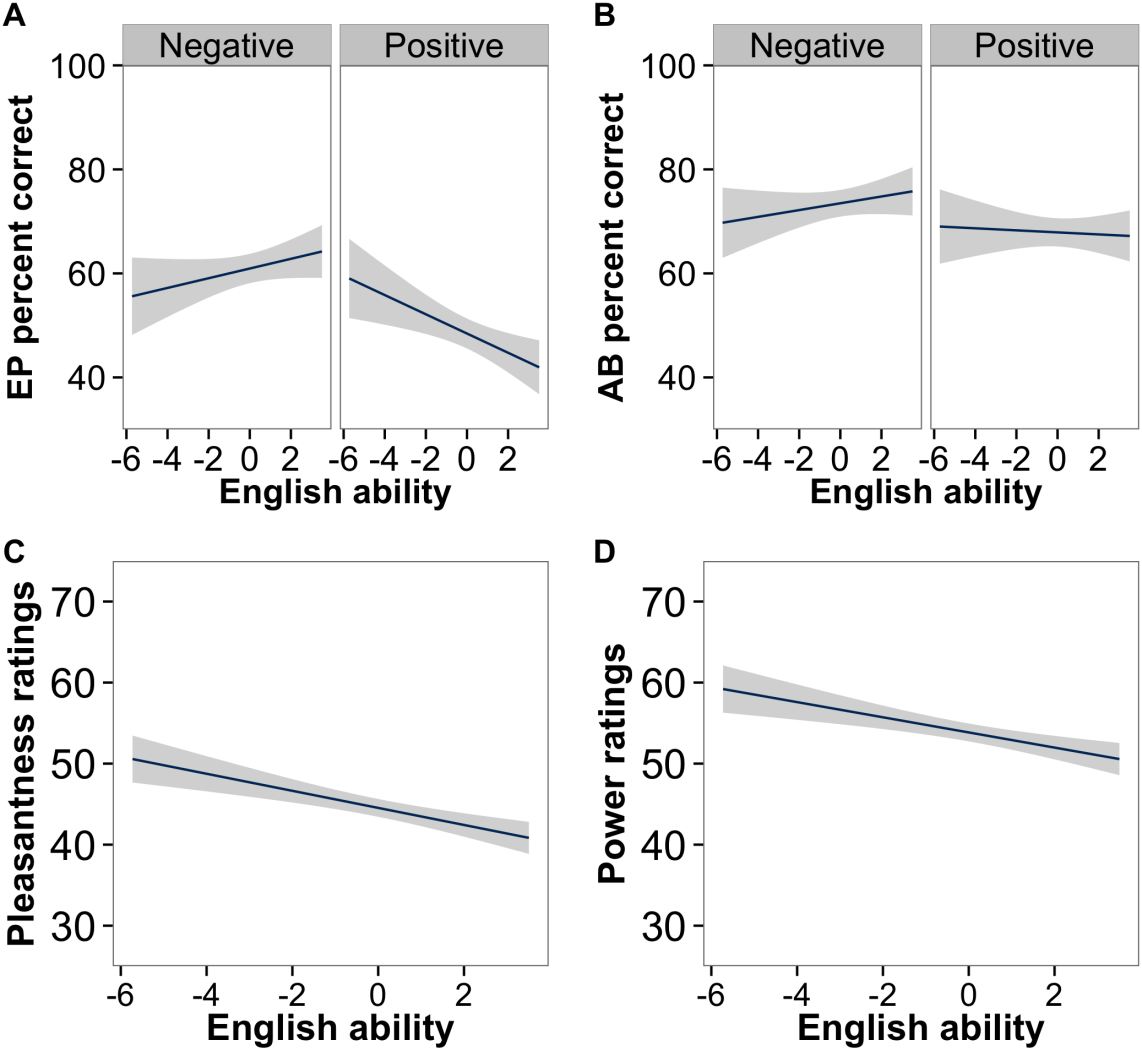
Linear regressions of percent correct responses by English ability for positive and negative emotions in the a) emotional prosody and b) affect burst conditions and of of c) pleasantness and d) power ratings by English ability in the emotional prosody condition. Shaded areas indicate standard deviations.

To explore the interaction between self-reported English ability and valence, the data was divided into positive and negative subsets and separate analyses were run on each subset with English ability as a fixed factor and a random slope by English ability for stimulus. For the positive emotions, there was a significant effect of English ability (*β* = -0.09, *SE* = 0.04, *z* = -2.41, *p* = .02), with a negative relation between English ability and proportion correct (i.e., higher English ability was associated with lower performance). For the negative emotions, English ability was not a significant factor (*β* = 0.06, *SE* = 0.05, *z* = 1.12, *p* = .26; see Fig 2A).

Analyses of the four continuous measures (not a dichotomous variable, so t-scores are reported) showed that for emotion intensity, the effect of valence was significant (*β* = -9.05, *SE* = 3.22, df = 49.9, *t =* -2.81, *p =* .007), with the negative emotions rated as more intense than the positive emotions, but no other factors were significant. For pleasantness, there was a significant effect of valence (*β* = -30.42, *SE* = 3.92, df = 59.2, *t* = 7.67, *p* < .001): the positive emotions were rated as more pleasant than the negative emotions. There was also a significant effect of English ability (*β* = -1.06, *SE* = 0.39, df = 53.2, *t* = -2.73, *p* = .009). Participants who were better in English rated the utterances overall as less pleasant (see Fig 2C). The interaction between valence and English ability was not significant (*β* = 0.25, *SE* = 0.72, df = 53.90, *t* = 0.35, *p* = .73). For power, there was a significant effect of valence (*β* = 13.9, *SE* = 5.19, df = 49.7, *t* = 2.67, *p* = .01). Participants rated the persons expressing the positive emotions as more powerful than the persons speaking the negative emotions. There was also a significant effect of English ability (*β* = -0.94, *SE* = 0.40, df = 46.1, *t* = -2.35, *p* = .02), with power ratings and English ability being negatively correlated (see Fig 2D). As with pleasantness, the interaction between valence and English ability was not significant (*β* = 0.03, *SE* = 0.55, df = 46.1, *t* = 0.05, *p* = .96). For activation, there were no significant effects (all *p*s > .27). See Table 4 for means and standard deviations.

**Table 4:** Mean ratings for the continuous measures for each valence.

	Positive	Negative
Emotional Prosody		
Intensity	56 (24)	65 (24)**
Pleasantness	60 (24)	29 (20)**
Power	61 (21)	47 (30)*
Alertness	66 (23)	65 (25)
Affect bursts		
Intensity	62 (24)	72 (22)**
Pleasantness	71 (22)	22 (20)**
Power	64 (19)	37 (25)**
Alertness	70 (23)	63 (25)*

Valences rated significantly differently at *p < .05 **p < .01

### Results of the affect burst condition

See Table 5 for a summary of the results. There was no main effect of English ability on the identification accuracy of the vocal affect bursts (*p* = .46), and English ability did not interact with any other factors (see Fig 2B). There was a significant main effect of valence (*p* = .007). The proportion correct was higher for negative (*M* = 0.73, *SD* = 0.44) than for positive emotions (*M* = 0.68, *SD* = 0.47). There was also a significant main effect for GEMEP score (*p* = .002) and a significant interaction between GEMEP score and valence (*p* < .001). No other effects in the model were significant (all *p*s > .20). Binomial tests showed that all emotions were identified more often than chance (see Table 3).

**Table 5:** Results from affect burst condition.

	*β* (Estimate)	Standard Error	*z*-value	Probability > |*z*|
(Intercept)	-0.60	0.65	-0.93	.35
Valence	2.82	1.05	2.69	.007
English ability	0.03	0.03	0.74	.46
GEMEP score	2.71	0.86	3.14	.002
Valence * English ability	-0.06	0.05	-1.24	.21
Valence * GEMEP score	-4.58	1.34	-3.40	< .001

To explore the interaction between GEMEP score and valence, subset analyses were performed as in the emotional prosody condition, with the fixed factor of GEMEP score, random intercept by subject, and a random slope of GEMEP score for stimulus. GEMEP score was positively correlated with performance for the negative emotions (*β* = 5.13, *SE* = 1.45, *z* = 3.55, *p* < .001), but was not correlated with performance for the positive emotions (*β* = -0.42, *SE* = 1.09, *z* = -0.39, *p* = .70).

As in the emotional prosody condition, linear mixed-effects models were run on the four continuous measures. For all four continuous measures, valence was the sole significant factor. As this is not relevant for the questions in the present study, these results are not discussed here, but see Table 6 for results, and Table 4 for means and standard deviations.

**Table 6:** Results from continuous measures analyses: Affect burst condition.

Valence factor	*β*(Estimate)	Standard Error	*df*	*t*-value	Probability > |*t*|
Emotion Intensity	-10.01	3.23	56.5	-1.10	.003
Pleasantness	48.8	3.58	79.5	13.6	<.001
Power	26.8	4.20	61.8	6.38	<.001
Activation	7.0	3.35	58.3	2.09	.04

## Discussion

This study was designed to answer three questions. The first was to what extent French speakers’ self-reported degree of L2 knowledge of English is related to their recognition and ratings of emotional expressions produced by American English speakers. Second was whether this relation would differ for emotional prosody vs. affect bursts, and third was whether the relation would differ for positive vs. negative emotions. For the emotion identification task, the answer to the first question was qualified by the answer to the second: there was no overall effect of English ability on identification accuracy, but there was an effect of English ability on identification of positive emotions in the emotional prosody condition. For the continuous measures, English ability was negatively correlated with both pleasantness and power ratings across positive and negative emotions in the emotional prosody condition.

### Identification of emotions

With increasing self-reported English ability, participants’ identification of positive prosodic expressions became less accurate. We speculate that the factors underlying this effect relate to an interference between the semantics present in the stimuli and the emotion. Participants with more English ability may have paid more attention than those with less English ability to the lexical and semantic aspects of the sentence. There was no effect of English ability on identification accuracy or ratings of the affect bursts, which supports this idea. If so, this top-down interference may have made it more difficult for them to accurately identify the emotions. One question raised by this explanation is as follows: why would there be an interference in the present study between semantics and emotion, when previous studies have not shown any such effect? This can be attributed to the paucity of previous research on L2 experience and positive emotion perception. As discussed in the introduction, previous studies examining L2 experience and emotion perception have included at most one unambiguously positive emotion (joy). With this positive emotion being only one of four or five being studied, it is unlikely that a positive-emotion-specific effect would have been statistically significant.

Why were positive and not negative emotions affected? As discussed in the introduction, recognition of negative emotions both within and across groups is likely more important to survival, and thus more likely to be evolutionarily important. Conversely, recognition of positive emotions is likely to be important within cultures for reinforcing social connections, but not necessarily between cultures. Thus, positive emotions may be less universal, and the way they are communicated is more likely to vary cross-linguistically. As such, positive emotions may be subject to a more complex interaction between the prosodic cues and the lexico-semantics of an utterance. Negative emotions, being communicated in a more universal manner, may have been more rapidly identified, leaving little opportunity for the lexico-semantic interference.

Varying difficulty among the emotions may also have played a role. In the emotional prosody task, visual inspection of Table 4 suggests that the negative emotions were in general easier to identify than the positive emotions, with the exception of disgust. This could be another reason why the effect of English ability was evident only for the positive emotions. Perhaps with increasing identification difficulty, increasing concentration is required, and interference from the lexical or semantic structure of the utterance is more likely to occur. Based on the logic discussed above, we argue that participants with better English would be more susceptible to this influence.

Out of previous studies examining the effect of proficiency on emotion identification in a second language, only Graham et al. [4] is comparable in methodology (i.e., participants all had the same cultural exposure, and differing levels of proficiency in a non-tonal L2 were tested). Like the present study, Graham et al. [4] also showed no effect of proficiency among their L2 groups of participants. Crucially, though, in the present study an effect was revealed when positive and negative emotions were examined separately. The only positive emotion tested in Graham et al. [4] was joy, so it is unknown if they would have found similar results. One previous study [7] did separately examine one positive and two negative emotions and found that L2 listeners performed less well than native listeners in their perception of positive and neutral but not negative emotions. These results, along with the present study, support the idea that positive and negative emotions are processed differently, and that positive emotions are processed in a more culturally or linguistically specific way. Together, these results suggest that the difference between positive and negative emotions needs to be a focus of future studies on emotional prosody.

### Continuous measures

There were significant effects of English ability on only two of the continuous measures—pleasantness and power—and only for the emotional prosody condition. Participants who considered themselves better at English rated the emotions overall as less pleasant and the speakers overall as less powerful. There were no interactions between valence and English ability, so these effects did not vary significantly between positive and negative emotions. As previously discussed for emotion identification, the effects of English ability on the continuous ratings could also be caused by the lexico-semantics interfering with or changing perception of emotion for participants with more knowledge of English. Alternatively, specific to pleasantness, it could be that participants with better English listen to the utterances in a more speech-like way than participants with less good English. The participants who are less good at English may listen more exclusively to the prosody, i.e., the melody of the utterance, and find that melody to be more pleasant. Some support for this idea comes from research showing that manipulating acoustic cues can have different effects on pleasantness ratings in music versus speech [25]. For example, the authors found that higher pitch increased the pleasantness in speech but decreased it in music. Hence, the way participants listen to utterances may change the way they interpret prosodic cues.

### Limitations and future directions

The present study used self-report measures of proficiency. These measures, although they have been extensively validated and are highly correlated with numerous proficiency measures [17, 19], are nonetheless subjective. Therefore, it could be interesting for future studies instead to use an auditory language proficiency test as a more objective measure.

A second limitation of the present study is the sample size and the distribution of linguistic ability, which was a random factor in our study. Continuous measures such as these have advantages for exploratory studies—notably, allowing us to measure a cross-section of the population without restricting the measure to the extremes. However, there are also advantages to creating discrete groups of extremes, i.e., a group with no knowledge of English and a group who is fluent. This would be a good future direction to explore, with larger groups of subjects. Similarly, a fruitful direction to explore in the future would be a balanced 2x2x2 design, for example including native French and English speakers, comparing L2 with monolinguals, and having all participants rate both French and English stimuli. Within this 2x2x2 design, a starting point could be speakers who are L2 vs. monolingual judging stimuli in both their native and second languages.

Specifically concerning the stimuli, there are several directions to explore in future studies. First, it would be valuable to sample more emotions across the affective circumplex of positive-negative valence and high-low arousal. For example, it could be useful to sample a wider variety of low-arousal emotions, such as serenity or boredom. Another aspect for future studies to explore is to compare responses when there is semantic content to when this content is removed (by filtering, for example). One explanation for the potentially counterintuitive results in this study is that there could be an increased interference between semantic and prosodic content for participants with higher English ability. Studies can be designed directly to examine the effect of semantic content on emotion recognition [26], which would provide evidence for or against this explanation.

## Conclusions

The present study examined the effect of language experience on the perception of vocal emotion expressions within a single cultural group. Results indicated that there are subtle effects of L2 experience on emotion identification, but these are limited to positive emotions. Increasing L2 experience interfered with identification accuracy for positive emotions rather than enhancing it. This could have implications for the social experience of L2 learners when communicating with others in their L2; increasing understanding of the L2 may be accompanied by a slight decrease in ability to understand subtle differences between positive emotions among other speakers.

## Supporting Information

S1 File Instruction sheet given to participants (translated from French to English). (DOCX)
