# Supplementary material for: Second Language Ability and Emotional Prosody Perception
Source: PLoS One. 2016 Jun 2;11(6):e0156855. doi: 10.1371/journal.pone.0156855 (PMC4890767; doi:10.1371/journal.pone.0156855)
Supplement: S1 File — (DOCX) [file pone.0156855.s001.docx]

For this first scale, your task will be to evaluate the intensity of the emotion that the speaker is expressing. Emotions can vary in intensity, for example, a person could be slightly angry (a bit irritated) or very angry (furious, raging). If you believe that the speaker is expressing a very high-intensity emotion, you should move the bar all the way to the right side of the scale. If, instead, you believe that he or she is expressing a low-intensity emotion, you should move the bar all the way to the left of the scale. Keep in mind that the intensity is not the same as the clarity of the emotion. A speaker could clearly express anger, but nonetheless be expressing a low-intensity anger (the person is clearly angry, but is only a bit irritated and not furious).
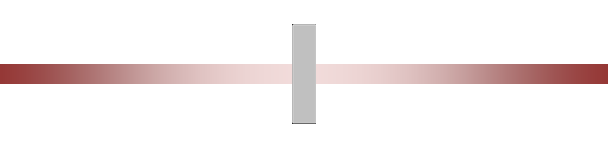


***Weak emotion*** ***Strong emotion***

On this second scale, your task will be to evaluate the pleasantness of the emotion expressed by the speaker. Some emotions can be more or less pleasant than others; for example sadness or anger can be more or less unpleasant. The same can be said for pride or joy, which could be more or less pleasant. If the emotion is pleasant, move the bar to the right. If, on the other hand, the emotion is unpleasant, move the bar to the left.


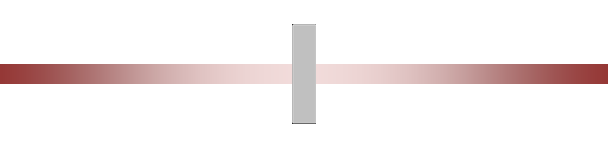


***Unpleasant emotion Pleasant emotion***

On this third scale, your task will be to evaluate the speaker's control of the situation (or power). If you believe that the speaker has total control of the situation, you should move the bar all the way to the right. If, on the other hand, you believe that the speaker has no control of the situation, you should put the bar all the way to the left. If you put the bar in the middle, that means the person is neither dominant nor submissive.


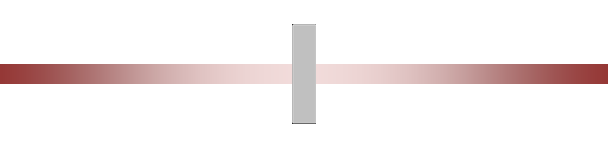


***Weak person Powerful person***

On this fourth scale, your task will be to evaluate the speaker's level of alertness or attentiveness. If you believe that the person is very alert, move the bar to the right, and if you believe that the person is not very alert (tired, sleepy) move the bar to the left.
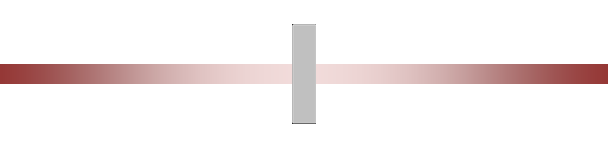


***Sleepy person Alert person***
